# Supplementary material for: Differences in prescribed medicinal cannabis use by cannabinoid product composition: Findings from the cannabis as medicine survey 2020 (CAMS-20) Australia-wide study
Source: PLoS One. 2024 Feb 14;19(2):e0297092. doi: 10.1371/journal.pone.0297092 (PMC10866492; doi:10.1371/journal.pone.0297092)
Supplement: Attachment — Submitted filename: 12.11_Cover letter - response to reviewer comments.docx [file pone.0297092.s001.docx]

The Editor 
PLOS ONE

November 12, 2023 

**Re: Revised manuscript – “Differences in prescribed medicinal cannabis use by cannabinoid product composition: Findings from the CAMS-20 Australia-wide study”**

Dear Editor,

Thank you for considering our manuscript, and for the opportunity to revise some aspects. I have addressed your feedback – in particular re patient content and patent declaration which I have added into the Methods (ethics) section and the competing interest section of the manuscript respectively. – This does not alter our adherence to PLOS ONE policies on sharing of data and/or materials.

There are no ethical/legal restrictions on sharing the dataset so we will upload the minimal anonymous data set needed to replicate our study findings. It can be found at this website: https://osf.io/f2yc3/

We also thank the reviewers for their comments and suggestions, all of which we have considered and addressed. Our responses to the reviewers’ comments are outlined below.

Thank-you for considering our revised manuscript.

Kind regards

Dr Benjamin Trevitt

|  | **Comment** | **Response** |
| --- | --- | --- |
|  | **Reviewer: 1** | |
|  | In this manuscript the authors have analysed the data they collected from the CAMS-20 survey of patients on their user experience in Australia who were taking a medicinal product (medicine) that contained the active ingredients of cannabidiol (CBD) and/or tetrahydrocannabinol (THC). This work is a follow-on from analysis already conducted and published on the CAMS-20 Study; reference 6 in this manuscript. I recommend acceptance pending the following points: | **Thankyou for your comments. Please see below for responses:** |
|  | 1. Lines 82 to 84. This statement about evidence for cannabis for non-cancer related pain cites TGA documents rather than research papers. I would recommend two recently completed Australian studies that looked at efficacy and safety of cannabis for pain as better references. (British Journal of Pain, 2023, 17,2 206-2017; and International Journal of Pharmacy practice, 2023, 31, 70-79). The second reference is relevant for the authors discussion of comedications in their manuscript (lines 239-242; 316-330) | **Thankyou. I have added in these recommended references.** |
|  | 2. In the introduction is a bit circle and repeats itself. For example lines 61-63 and 93-94 cover similar information. The introduction should be revised to make it more streamlined. | Thankyou. I have removed the sentence in lines 61-63. I have also made some other cuts/edits to make the introduction more streamlined. |
|  | 3. It is not clear what the inclusion criteria for the subgroup of the 546 participants was (line 117). I’m guessing that it was any participant that took a legal form of cannabis (implied by line 137) and participants with missing data (line 151) but that is ambiguous and should be made more clear at line 117 | Thankyou. I have clarified this at the start of the methods section [see lines 119-125 of the marked version] |
|  | 4. Line 151. What constituted "missing data". How much data had to be "missing" before it was excluded? | Thankyou. I think I have made this clearer in lines 119-125. In short, as long as the participants responded and said they were using some form of prescribed medicinal cannabis AND they could report the constituents of the prescribed medicinal cannabis AND said constituents remained consistent week to week, they were included in the study (546 participants met this criteria). If participants did not respond to other questions, they were still included in the overall data analysis, but were treated as missing in the relevant subgroup analyses. |
|  | 5. Details of the statistic tests undertaken, and not just the software, need to be given (line 163). | Thankyou. See lines 164-177 in the newly uploaded version. Please let me know if further information is required. |
|  | 6. The research team put products into three categories TO, T+C and CO, but the TGA defines five categories of products. Would it not have been better to evaluate the products based on the TGA categories? Particularly when later (lines 180/181) the terms “mostly THC” and “mostly CBD” products are used. | We merged together only THC and mostly THC ; and only CBD and mostly CBD; as otherwise the size of the groups would have been too small. For example, if we had used the 5 TGA categories, the THC only” group would have only been of size 20. |
|  | 7. “Not sure if I agree with the statement (line 183): TO group participants were significantly younger than T+C and CO group participants. The average age of TO was 42.5 and average age of the others is 46.3 and 47.5. Those are very similar ages. I think the authors mean there was a statistical difference in their average ages, not that they were significantly younger. If this is changed here, then it also needs to be changed in the abstract and discussion (line 265).” | Thankyou, I have changed the wording to reflect this comment. |
|  | 8. On what basis do the authors make the following statement about employment status (lines 184 to 186): “Additionally, when compared with CO users, TO users were more likely to be male, unemployed, and have regularly used illicit cannabis in the past” given their table finds more statistical difference between any of the three categories. | Thankyou. I have adjusted this sentence to reflect there is no difference in employment status between the three categories. |
|  | **Reviewer: 2** | |
|  | 1. The article by Trevitt et al describes the results of a survey of medicinal cannabis users, this is a well written article highlighting that major benefits of medicinal cannabis come from predominately THC containing products and not CBD products | Thankyou for your comment and for reviewing the article. |
|  |  |  |
| **Reviewer: 3** | | |
| The submission by Mr Benjamin Trevitt and colleagues presents findings from a cross-sectional study of prescribed medicinal cannabis users in Australia that sought to better understand use of different product compositions and formulations, cost of product use, and self-reported change in symptoms associated with the primary medical indication for use over time. While several published cross-sectional studies have examined similar questions, the novelty of the current study is its focus on medical cannabis use in Australia, a county where medical cannabis is legal and adult-use cannabis (recreational) is illegal. Participants were assigned to one of three groups based on their provided medical cannabis formulation (THC, THC+CBD, and CBD). Converging with studies published in other countries, THC products were endorsed more frequently among young male users with inhalation as the preferred route of administration. Compared to participants using CBD products, participants spent more on THC products and these same individuals self-reported improvements in the conditions/symptoms for which they were prescribed medical cannabis. The manuscript is concise, organised, and well-written. Below, please find my comments and feedback addressed to the attention of the authors. | | Thankyou very much for your feedback. Please see below for responses to your concerns |
| 1. Title: The authors should consider filling out the full name of the survey study in the title before using an acronym (CAMS-20) | | Thankyou. I have addressed this. |
| 2. Abstract: For clarity, it would be helpful if the authors specified that the participants are Australian for both the Methods and Results sections of the abstract. The authors should also consider including the study’s country of origin in the conclusions section of the abstract, which sets this study apart from existing cross-sectional work published in other countries | | Thankyou, I have added this in to both the methods, results, and conclusion sections of the abstract. |
| 3. Background (General): The focus on Australia is important because it builds a knowledge base that other countries can refer to when considering reforms to cannabis legislation. Thus, it may be important to describe the current legal status of medical and non-medical cannabis in Australia for additional context. | | Thankyou for this comment. I have added some more information into the introduction |
| 4. Background (cont.): (Page 4, lines 55-56): Please describe what is meant by “…pro-MC scheduling recommendation” | | Thankyou – I have clarified this in the introduction: “recommendation that cannabis and cannabis resin be deleted from Schedule 4 of the single convention on narcotic Drugs” |
| 5. Background (cont.): (Page 4, line 57): Since the location (Australia) is the setting for the study, readers in other countries need a few more details that explain the purpose of the TGA and SAS-B | | Thankyou – I have added in some more background information into the introduction. |
| 6. Background (cont): (Page 5, line 86-91): To distinguish the current work from existing findings, the authors should consider specifying that a “…better understanding of MC product composition is critical….” among prescribed medicinal cannabis users in Australia. | | Thankyou – I have added this into the introduction. |
| 7. Background (cont): (Page 5-6; lines 94-105): Importantly, the authors state how it’s important to understand how medicinal cannabis is being used in the Australian context. Study location aside, a number of cross-sectional retrospective and prospective survey studies have been published in other countries, which raises the question of the value of the current work. It may be useful to tighten up the rationale/argument for conducting the current study earlier in the background. | | Thankyou, I have added in a section to the introduction which better illustrates the rationale for the study; in both the Australian context but also globally. |
| 8. Background (cont): (page 6, lines 107-112): The authors present the results of analyses that examined concurrent medication use, but this is not listed in any of the study’s three aims (but discussed in the context of survey questionnaire content). | | Thankyou, I added a fourth aim at the end of the background section ( “(4): to review safety profiles of prescribed cannabinoid products in terms of concurrent medication use and driving patterns post cannabinoid product consumption”) |
| 9. Methods: (page 6, lines 117-119): Were the authors able to verify legal prescribed medical cannabis use or was it based on participant self-report | | It was based on participant self-report, and unfortunately not able to be verified. |
| 10. Methods: (page 7, lines 130-135): Please describe how study participants were recruited. Was any financial remuneration given to participants? The authors refer the reader to an existing publication, but these two details can be described concisely here. | | Thankyou, I have added in how the study participants were recruited. There was no financial remuneration |
| 11. Methods: The current abbreviations for each of the three groups (TO, T+C, CO) could be improved for greater clarity. For example soulc these be changed to THC, THC+CBD, CBD or something similar? The current abbrevations are not that intuitive. In this same section it would be helpful to specify the sample size for each of the three groups. | | Thankyou. I have changed these abbreviations to something clearer + have added in sample sizes to the three groups. |
| 12. Were any methods to handle missing data considered? | | Missing data was excluded from analysis as there was minimal missing data, and thus there was no reason to think it would have impact on the findings.  Only 19 clients on medicinal cannabis were not able to determine the constituents of their cannabis and only they were excluded/treated as missing. |
| 13. The data presented in each table seems to indicate that comparisons were made using more than 50 separate variables. The authors used bonferonni’s procedure to correct for multiple comparisons. It would be helpful to include a bonferonni adjusted p value column in addition to standard p value column for the sake of interpretation | | Thankyou. Each outcome variable in the tables was considered as continuous/binary in their own right. For example in table 2, row 1: main reason for use as pain was treated as a binary outcome (YES/NO), separately from MHSU etc. The p value in the column is already the bonferroni’s adjusted p value. Bonferonni’s adjusted p values were calculated in cases where >2 groups were being compared (i.e. in the tables with THC only vs THC+CBD vs CBD only as predictive covariates). I have added in a standard p value to these tables; and labelled the bonferonni’s p value as such to avoid confusion. |
| 14. How did participants complete the survey? Computer? Tablet? Mobile? All of the above? | | Data was collected online through RedCap, a secure web based application |
| 15. Results (Page 9, Line 172): Please indicate total number of partiicipants that were screened for the study and provide the survey completion rate | | Of the 2152 respondents who commenced the survey, 601 reported using prescribed medicinal cannabis. Data was excluded for 55 participants who either did not indicate the composition of the prescribed MC or who stated that the contents varied between batches. Data are reported for the remaining 546 respondents. Overall, the survey of this subgroup had a 80.2% completion rate |
| 16. Results (Page 9-10, lines 183-94) Please provide the results of the statistical tests and the p-values for comparisons when discussing the results in the main text | | Thankyou, I have added these to the results |
| 17. Results (page 10, lines 191-192): Please specific the currency that responds to the amount participants spend on medicinal cannabis products | | Thankyou. It is in Australian dollars. I have amended this |
| 18. Results (Page 13, lines 202-210) Please provide the results of the statistical tests and the p-values for comparisons when discussing the results in the main text | | Thankyou, I have added these to the results |
| 19. Results (Page 13, 204): For patient reported conditions involving mental health and substance use, are the authors able to extract any specific mental health conditions (anxiety, depression, PTSD) in the current sample, or was mental health and substance use a broad category | | We reported some mental health and substance use (MHSU) as a broad category, as well as the more common subgroups of mental health conditions, namely anxiety and depression – as these were the most common mental health conditions. Other less common mental health conditions were asked about (e.g. bipolar disorder, however their prevalences were very low (2 in the case of bipolar disorder). |
| 20. Results (page 16, lines 214-218): The authors might consider ordering the side effects listed in table 3 in order of reported prevalence (e.g. highest to lowest). Please also report p values in this section | | Thankyou, we have amended this. |
| 21. Results (Page 16 , lines 223-227): It would be helpful to briefly detail the psychometric properties of the PGIC and the complete wording of the question that required partipcants to rate symptom improvement. Also, what is the time window that participants are using to report on their symptoms. | | Thankyou I have added this into the results  The time window participants are using re improvement in symptoms is the past 12 months. I have also added this in. |
| 22. Results (Page 18, lines 239-246): Please include relevant test statistics and p values | | Thankyou, we have amended this |
| 23. Results (Page 19, lines 250-257): Please include relevant test statistics and p values | | Thankyou, we have amended this. |
| 24. Discussion (Pg 19, lines 263-264): It may be useful to briefly state the current legal status of medical and non-medical cannabis in Australia | | Thankyou, I have added this in |
| 25. Discussion (Pg 20-21, lines 272-299): The authors discuss the methodological barriers of previous studies but don’t discuss here whether the current study had any major limitations. | | Thankyou, I have rearranged paragraphs to address this. |
| 26. The location of table 7 should be moved out of the discussion section. | | Thankyou. I have moved the table into the results |
| 27. (page 23, lines 346-353): The conclusding paragraph doesn’t include any instance of the word Australia which is the major element of how this study differs from existing work | | Thankyou. I have added this in. |
| **Reviewer: 4** | | |
| Thankyou for the opportunity to review this important study manuscript. Overall, as the first study of its kind in the Australian context, this is an important body of work in furthering the understanding of patterns of MC use and prescribing amongst clinicians here.  I have no major concerns and overall would recommend this study for publication. I do however note one limitation that has largely not been addressed in the results or discussion | | Thankyou for you feedback. Please see below where I have addressed your concerns |
| The study cohort consists of 546 people from the CAMS-20 data-set who report being prescribed MC products within the last year. The analyses rely on grouping these participants into 3 groups based on reported product composition. It is noted by the authors in the methods that 350 of these participants reported using both prescribed and illicit MC. The composition of the latter as acknowledged by the authors Is difficult to ascertain with clarity. It is not cleaer in the remainder of the manuscript how this issue has been dealth with the reported results and discussion. Further detail would be discussed and would replace the results in context. | | Thankyou for this comment:  Questions regarding   - Medicinal cost - Route of administration - Frequency of use - Main reason for use - Degree of improvement   Were all asked of prescribed and illicit medicinal cannabis separately (so all the questions related to the above were asked specifically about prescribed MC and then asked again specifically about illicit MC – so some people did answer it twice). Thus the data in this paper related to the above questions has been asked and analysed specifically about prescribed MC (I have added some more information into the paper, particularly the methods section, such that this is clearer)  However questions related to driving + side-effects were not separated in terms of prescribed or illicit. We reran the analysis without the dual users (i.e. only with those who said they used prescribed cannabis only) and the substantative conclusions were unchanged. Here we found the following:   - The most common side-effects were still dry mouth, drowsiness and fatigue; and those on THC were statistically more likely to get dry mouth (OR = 2.3, (1.23-4.22), p<0.001) and drowsiness (OR = 3.2, (1.71,6.1), p<0.001) than those on CBD. - Again, no statistically significant differences were found in likelihood of THC vs CBD driving within 24, 13-14, 7-12 or 4-6 hours. Borderline difference for 1-3 hours was noted; and like we found previously, statistically significant differneces noted for likelihood of driving within 1 hour.   Given the simarilty of the two findings, we decided to report the results of the original analysis. |
|  | **Reviewer: 5** | |
|  | Abstract requires some additional information for clarity. Clarification that those using CBD dominant products were preferred by older individuals, non-inhaled and more females? What indications was CBD use reported for? Where would balanced 1:1 types be considered? What about people using multiple product types? There is lack of detail on efficacy report measures, do you mean patient-reported effectiveness? When were these measured? Side effect profiles required quantification. Drug interactions is mentioned in the conclusion but there is no data presented in the abstract to support this statement. | Thankyou, I have made amendments to the abstract to address these concerns |
|  | Article comments: Some of the jurisdictional nuances re S4, S8 products require definition to improve clarity to an international audience | Thankyou, I have added this in. |
|  | Article comments: 601 reported prescribed medical cannabis in the initial survey and 546 were included as a subgroup, if that means that 55 people were using illicit sources only? what is the rationale for excluding their experiences? If it was just that they did not report complete data mention that those without complete or unclear on product composition data were excluded. | I have tried to make this clearer in the paper.  But in short there were 1600 people who answered the survey. Of them, 601 reported using either prescribed MC or both prescribed and illicit MC. We excluded the group using illicit only MC as we thought it would be very difficult to be sure of the composition of their MC |
|  | Article comments: How were patients using multiple cannabis products analysed? | They were excluded from the study |
|  | Article comments: The groups based on composition would be more useful if reclassified to align with the TGA cannabis product categories | Unfortunately, we merged together only THC and mostly THC ; and only CBD and mostly CBD; as otherwise the size of the groups would have been too small. For example, if we had used the 5 TGA categories, the THC only” group would have only been of size 20. |
|  | The rationale for grouping TO and T+C is not well established, these patients could be taking balanced products with high amounts of CBD, as such contributing to both side effects and self-reported benefits | Thankyou for this comment, and I have added some information into the paper to address this. The relative potency of THC (usually used in doses of up to 30-40mg per day) compared to CBD (usually requiring doses of 300-400mg plus for efficacy for most conditions) means that most patients being prescribed a THC / CBD product are getting doses close to 1:1, and hence, the CBD effects are unclear. Also, to our understanding, high CBD + low THC is not a common comibination. However we do appreciate the comment and have added this to our limitations. |
|  | The side effects table 3 is mostly THC related side effects so not seeing these with the CBD only group is logical. What would be more relevant and interesting would be to look at the differences between category 2-5 products – do side effect profiles change with CBD+THC vs THC alone? | Unfortunately, we merged together only THC and mostly THC ; and only CBD and mostly CBD; as otherwise the size of the groups would have been too small. For example, if we had used the 5 TGA categories, the THC only” group would have only been of size 20. |
|  | Line 206-208 should be reworded as it currently states no differences but also differences. What are general presentations? | I have reworded this, thankyou. By general presentations, I was meaning ‘big picture’ presentations (e.g. pain) as opposed to specific presentations such as back pain or nerve pain. |
|  | Table 6 – this data should be separated out THC only or THC+CBD? This data may be more clinically interesting re: potential entourage effects Was driving data different amongst acute and chronic exposures? | Unfortunately we do not have sufficient data to determine ‘chronic’ vs ‘acute exposure.  We decided to group THC and THC+CBD together given the fact in Australia that any driving with any THC onboard is illegal. |
|  | Line 277-280 should be reworded to exclude drug resistant epilepsy which has high quality evidence. | Thankyou I have added this in |
|  | If possible, add “in Australia” to the short title Thorough editing as there are some extra periods and spacing in places | Thankyou, I have addressed this. |
| **Reviewer: 6** | | |
| This is an interesting and well-written paper. The survey method and analysis are largely appropriate to the research questions being asked. Conclusions are stated in a careful and mostly balanced way. I enjoyed reading it. However, I do have some concerns about the survey design and analysis that ought to be addressed. | | Thankyou for your review. Please see below for responses to your comments. |
| 1) Most of the subsample analysed here used both prescribed cannabis AND illegal cannabis for therapeutic/medicinal reasons, but I am not sure what measures referred to prescribed MC and can be attributed specifically to those products. The methods section does not specify e.g. if self-reported improvements in symptoms or side-effects were asked with explicit reference to prescribed MC? This may be a problem if a participant reported use of prescribed CBD-only, but also used THC from the illegal market... I would expect that the side effects/improvements would be different from CBD-only vs. if they answered the question considering ALL types of cannabis they used for therapeutic reasons. This also applies to other measures, e.g. driving after use of ANY product, or was the questions explicit and referred to driving after use of prescribed products? | | Thankyou for this comment. I have re-addressed this in the paper, but in short:  Questions regarding   - Medicinal cost - Route of administration - Frequency of use - Main reason for use - Degree of improvement   Were all asked of prescribed and illicit medicinal cannabis separately (so all the questions related to the above were asked specifically about prescribed MC and then asked again specifically about illicit MC – so some people did answer it twice). Thus the data in this paper is related to the above questions has been asked specifically about prescribed MC (I have added some more into the report, particularly the methods section, such that this is clearer)  However questions related to driving + side-effects were not separated in terms of prescribed or illicit. We reran the analysis without the dual users (i.e. only with those who said they used prescribed cannabis only) and the substantative conclusions were unchanged. Here we found the following:   - The most common side-effects were still dry mouth, drowsiness and fatigue; and those on THC were statistically more likely to get dry mouth (OR = 2.3, (1.23-4.22), p<0.001) and drowsiness (OR = 3.2, (1.71,6.1), p<0.001) than those on CBD. - Again, no statistically significant differences were found in likelihood of THC vs CBD driving within 24, 13-14, 7-12 or 4-6 hours. Borderline difference for 1-3 hours was noted; and like we found previously statistically significant differneces noted for likelihood of driving within 1 hour.   Given the simarilty of the two findings, we decided to report the results of the original analysis. |
| 2) Need more clarity about the definition of prescribed MC consumer. I.e. is ONE prescription in the past 12 or 6 months enough to qualify? Again, this is an issue as most participants in this analysis used both legal and illegal products; so it is possible they may be “mostly illegal MC consumers with only one MC prescription”. Did the survey asked whether they actually bought and used the prescribed product (or is receiving a prescription enough?). We know from other similar jurisdictions that some patients may request prescriptions simply to have it on hand in case of legal problems. | | To be classified as a prescribed medicinal cannabis consumer, one had to either have used their own prescribed medicinal cannabis in the past 2 weeks and plan on using it in the future (~87% of prescribed MC users were in this category); or have used it regularly in the last 12 months but stopped >2 weeks ago without plan of continuing (~8% of prescribed MC users were in this category); or occasionally use prescribed MC when required and plan on continuing to do so in the future (~4% of prescribed MC users were in this category). I have added this in the methods section for more clarity. |
| 3) Route of administration (ROA) is mentioned in the analysis but not in the methods section. It would be good to get more clarity about the measures collected and which questions explicitly referred to prescribed MC. Regarding ROA, I can only see vaporizer and inhaler ROA in Table 1 – others not included? | | Thankyou for this comment. I have added in some details into the methods section; re which questions referred specifically to the prescribed MC.  There were a number of subgroups in the questionnaire but they fell into three primary groups (oral (e.g. tablet, edibles, oil, spray); inahled (e.g. joint, pipe, bong); vaporiser; suppository and topical (e.g. cream).  However topical + suppository had only 2 and 1 participant each respectively and hence were excluded. Oral is the reference group here. |
| The points above have significant implications for the conclusions we can draw from the analysis. I appreciate it is impossible to go back and ask more explicit questions linked to prescribed MC at this stage. The issues above ought to be addressed and controlled for, or discussed in the limitations. Overall, I enjoyed reading the study and I think this will be a useful contribution to the literature if authors are able to address the points above. | | Thankyou for your comments! (See above) |
|  | |  |
